# Unraveling forensic timelines using molecular markers in *Phormia regina* maggots

**DOI:** 10.1371/journal.pgen.1011948

**Published:** 2025-12-04

**Authors:** Sheng-Hao Lin, Anthony J. Bellantuono, Kristian Lopez, Jeffrey D. Wells, Matthew DeGennaro

**Affiliations:** 1 Department of Biological Sciences, Florida International University, Miami, Florida, United States of America; 2 Biomolecular Sciences Institute, Florida International University, Miami, Florida, United States of America; 3 Global Forensic and Justice Center, Florida International University, Miami, Florida, United States of America; Michigan State University, UNITED STATES OF AMERICA

## Abstract

In the medico-legal application of forensic entomology, estimating the time of death is critical and traditionally relies on changes in observable traits of carrion feeding insect larvae. Traits such as size, weight, and morphology can be used to predict the insect specimen age and help define the minimum time since death. The blowfly *Phormia regina* Meigen (Diptera: Calliphoridae) is a key forensic insect, yet age estimation for older maggots in this and other carrion-feeding species is particularly challenging due to the limited morphological changes in the late-stage larvae. To enhance age-estimation precision, we employed transcriptomic profiling on blowfly maggots, aiming to identify genes as markers for time of death estimation. Our study characterized maggot development, reinforcing that weight and behavior cannot precisely determine age between 100 and 130 hours at 27.5 °C. We built a chromosomal scale annotated genome, establishing a reliable database for uncovering transcriptomic signatures during larval development. Applying differential gene expression analyses, weighted gene co-expression network analysis, and the generalized linear model, we identified nine candidate genes (*y5078*, *y5076*, *agt2, ech1, dhb4, asm, gabd, acohc, ivd*) that delineate the age of otherwise indeterminate maggots. This research introduces a molecular approach to address a longstanding problem in forensic entomology and promises to increase precision in determining the time of death at a crime scene.

## Author summary

In forensic entomology, estimating the time of death often depends on insect morphological changes. Like other blowflies, *Phormia regina* is a key species for postmortem interval estimation, but aging older maggots in the third larval instar phase is difficult due to minimal changes in observable traits. To improve accuracy, we used transcriptomic profiling to identify genetic markers for time of death estimation. Our study showed that weight and behavior are not reliable indicators of age in maggots 100–130 hours old. We developed an annotated genome to identify transcriptomic signatures during larval development. Using three distinct statistical analyses, we identified nine genes (*y5078*, *y5076*, *agt2*, *ech1*, *dhb4*, *asm*, *gabd*, *acohc*, *ivd*) that can determine the age of late third instar maggots. This molecular approach aims to enhance precision in forensic entomology.

## Introduction

Blowflies, members of the Calliphoridae family, are attracted to decaying organic matter like corpses by their keen sense of smell and sensitivity to decomposition cues [1]. Adult females typically deposit eggs on or near carrion, the larval food. These eggs hatch within hours to a few days, depending on environmental conditions, giving rise to maggots that progress through three larval stages before metamorphosing into adult flies. Knowledge of development rate under known environmental conditions, particularly temperature, is used to estimate the age of a specimen associated with a corpse. Since blowfly eggs are generally deposited on a cadaver after death, the age of the insect provides a critical minimum postmortem interval (PMI) estimate [2].

Estimating PMI is a common step in a death investigation [2]. Although there have been methodological improvements to PMI estimation using many disciplines including microbiology [3], biochemistry [4], anthropology [5] and chemistry [6], the task is still very challenging because decomposition rates are a function of many variables such as ambient temperature conditions and the effects of drugs in decomposing tissues [7]. Forensic entomological approaches are therefore key metrics to help establish time of death that is largely independent of body decomposition rates [8].

The most common insect analyzed from a death scene is a blowfly [9]. The life cycle of a blowfly includes egg, three larval (maggot) instars, prepupa and pupa (both formed within the last larval exoskeleton, the puparium), and imago (adult) [10–12]. Unlike some other insects, blowfly maggots can become committed to metamorphosis very early during the final larval stage [11]. Therefore they can pupate and form a functional adult at a size much smaller than maximum, which appears to be an adaptation to the intense competition for food between both invertebrate and vertebrate animals that can occur on carrion [13]. In this investigation, *Phormia regina (P. regina)* was used as the study species. *P. regina* is common across North America except south Florida, often peaking in density during spring and fall [14–16].

The standard approach in forensic entomology for age estimation of immature maggots relies on the change of body length, weight, and instar [17]. During development larval size increases steadily [18]; about midway through the third larval instar, larvae stop feeding and wander in search of a location to pupate [19]. During most of the third larval instar the size of a larva does not change or may decrease slightly. As a result, neither instar nor size can be used to distinguish ages within about 50% of the larval life stage of *P. regina* and other carrion-feeding blowflies.

Age estimation methods based on changes in gene expression levels have been used in pupae of the muscid fly *Hydrotaea spinigera* [20], the blowfly *Lucilia cuprina* [21] and the flesh fly, *Sarcophaga peregrina* [22]. Despite the need, age-related genetic markers of third instar blowfly maggots have not been characterized at a resolution that can improve post-mortem interval determination. Some forensically important blowflies including *Cochliomyia macellaria* [23], *Lucilia sericata* [24,25], *Chrysomya rufifacies* [26], *Aldrichina grahami* [27] and *Chrysomya megacephala* [28,29] have shown that genetic markers can be associated with maggot age, but higher resolution analysis of third instar maggots over this critical developmental period where physical differences cannot distinguish maggot age is required. In addition, the sampling in these studies was not done in short time intervals. This limits the ability of the molecular markers found to delineate specific age estimations during the long third instar.

In this study, we utilized transcriptome profiling to identify candidate genes to enhance the accuracy of blowfly maggot age estimation. We conducted a developmental study of *P. regina* maggots during their third larval instar. This blowfly species was chosen for its forensic importance across most of North America [30]. To support our investigation, we assembled a chromosomal-scale genome of *P. regina* as a robust reference for gene expression analysis and candidate gene identification. Additionally, we integrated Iso-Seq data to enhance genome annotation by sequencing full-length transcripts, which improves accuracy by enabling the direct detection of exon-intron boundaries [31]. We identified differentially expressed transcripts in maggots at 10-hour intervals during the third larval instar. We also found transcripts associated with maggots’ shift from feeding to wandering behavior. The candidate genes could be used to improve the accuracy of time of death determination.

## Results

### Identification of key transitions during maggot development

**Fig 1:**
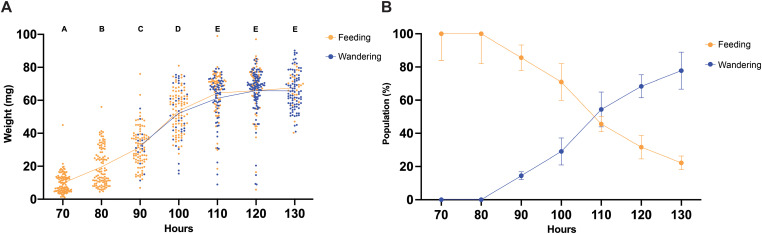
Tracking maggot weight and behavior from 70 hours old to 130 hours old. (A) Scatter plot of individually weighed maggots scored for feeding and wandering from 70 to 130 h. The straight line is the mean weight at each age cohort. The graph shows change in larval weight over four replicate experiments (n = 823). Brown-Forsythe and Welch ANOVA tests were performed on the weight of aging cohorts (*P* < 0.0001). (B) The mean weight of feeding (yellow) and wandering (blue) maggots from 70 to 130 h. Population transition between feeding and wandering maggots occurred between 100 ~ 120 h. The graphs showed the number of maggots scored as feeding or wandering (a) over four replicate experiments (n = 823). The error bars indicate the standard error of each cohort.

Maggot developmental changes over time are used to determine when a blowfly laid eggs on a corpse. We placed *P. regina* maggots of known age (~24 h) on chicken liver (S1A Fig). To determine changes in phenotype with age, maggots were reared and sampled without replacement. Maggots were observed to be feeding if they remained on the liver substrate and were defined as wandering if they had ventured away from the cup, settling in the sawdust at the base of the rearing container (S1B Fig). We found a steady increases in larval weight and size until 100 hours old (h). From ages 110 h to 130 h no discernible changes in weight or size were detected, highlighting the need for molecular determination of age for older maggots. The first appearance of wandering maggots was observed at approximately 90 h, with this behavior becoming predominant by the 130 h mark (Fig 1A and 1B). To compare maggot weights across multiple time points, we used Brown-Forsythe and Welch ANOVA tests, which are one-way ANOVAs that do not assume equal variances among groups. These tests assessed whether mean weights differed across developmental stages, our result showed increased weight between 70 and 100 h for both feeding and wandering maggots but there was no significant weight change between 110 and 130 h (Fig 1A and 1B). For pairwise comparisons between feeding and wandering maggots, we used the Mann–Whitney U test, a non-parametric test that assesses differences in median values between feeding and wandering groups when the assumption of normality is not met. Our results showed that the median weight of wandering maggots (66.10mg, n = 307) is significantly higher than that of feeding maggots (34.55mg, n = 507) (S2 Fig). The transition from feeding to wandering also suggests a potential developmental shift in gene expression, indicating that investigating the gene expression profiles during larval development could yield valuable markers to enhance the precision of time since death estimation.

### High quality *P. regina* genome assembly showed six pseudohaploid chromosomes

**Fig 2:**
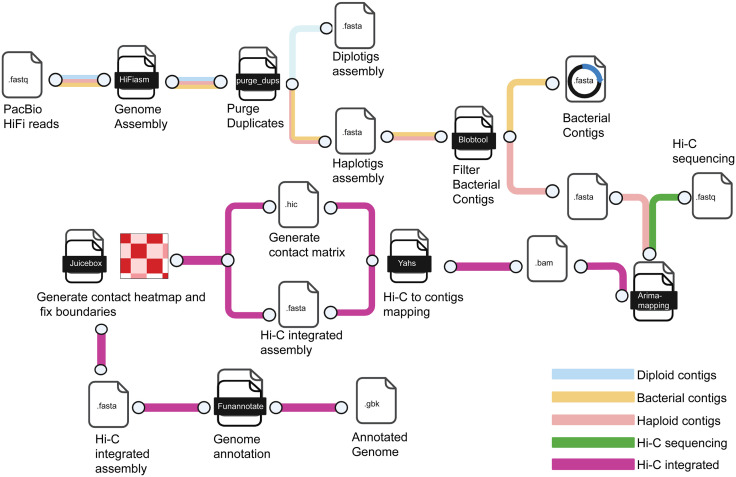
Genome assembly pipeline workflow. PacBio HiFi reads were assembled and duplicate sequences were purged. After the filtration of bacterial contaminant contigs from the assembly, the Hi-C sequences were mapped to the clean contigs for generating contact matrix and contact heat map. Created in BioRender. Lin, **S.** (2025) [32].

**Fig 3:**
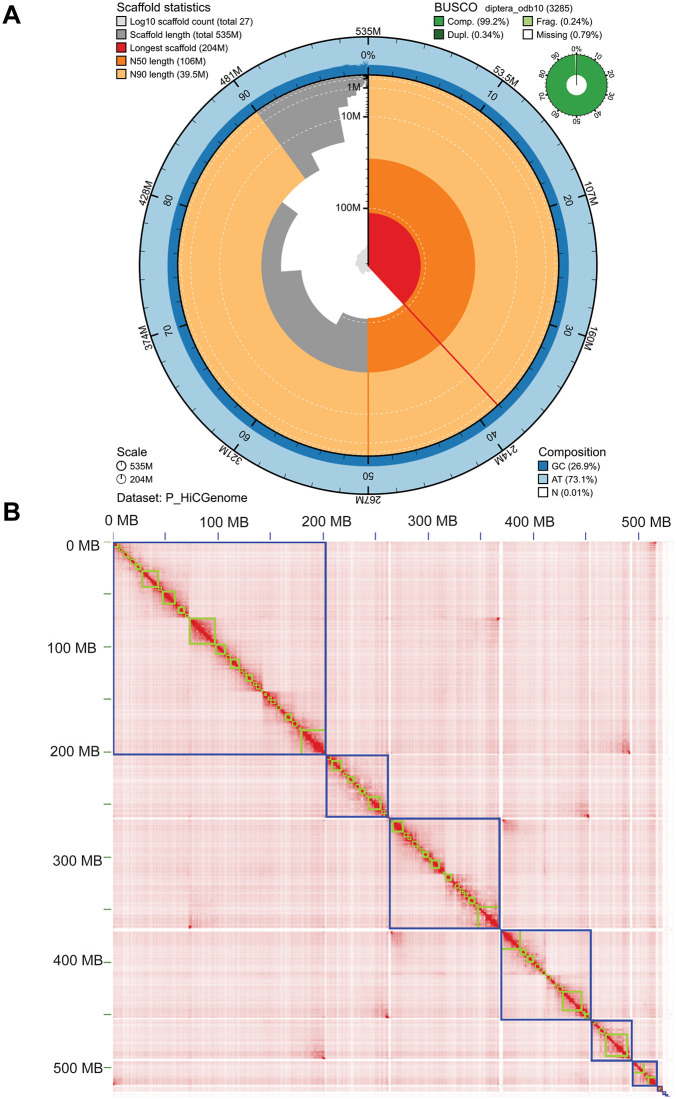
Chromosomal level genome assembly shows 6 pseudohaploid chromosomes in *Phormia regina.* **(A)** Snail plots summarized the BUSCO, AT & GC content and scaffold statistics of *P. regina* genome assembly without proteobacteria contigs. Red segments represented the size of scaffold; dark and light orange segments indicated N50 and N90 values; central light gray indicated the cumulative scaffold counts under order of magnitude; blue and light blue indicate the proportion of AT & GC content. Upper right corner showed BUSCO completeness score against the Diptera database. **(B)** Hi-C contact matrix heat map of the chromatin interactions and scaffolding compartments. These six pseudohaploid chromosomes were clearly delineated by blue boundaries, with red signals indicating high-frequency chromatin interactions within chromosomes. (Blue: Chromosomes, Green: scaffolds).

Prior to analyzing gene expression during larval development, we generated an updated, high-quality genome assembly of *P. regina* to serve as a reliable reference for transcript identification. Clean draft contigs were scaffolded using Hi-C data, and contact matrices were constructed to visualize chromatin architecture (Fig 2; see **Materials and methods**). Genome annotation was performed using the Funannotate pipeline, enabling the identification of protein-coding genes, including transcripts relevant to developmental stages. Assembly quality was assessed using a snail plot, which showed a contig N50 of 7.01 Mb and a total genome size of 530 Mb (**Fig 3A**). BUSCO analysis revealed a high completeness score with a low percentage of fragmented or missing orthologs, confirming the robustness of the assembly for downstream transcriptomic analyses. The plot also highlighted a low duplication rate among BUSCOs. The Hi-C contact heatmap revealed six well-defined chromosomal territories, consistent with the known karyotype of *P. regina* [33], supporting our chromosomal-level scaffolding (**Fig 3B**). Comparison with the previously available genome shows that the Hi-C-integrated assembly significantly improved genome contiguity, BUSCO completeness, and exon annotation, thus providing a robust genomic framework for transcript identification and expression profiling in *P. regina* (**Table 1**) [34]. The size of Hi-C integrated assembly (534 MB) is also similar to the average estimate (517 ± 2.5 MB) of male *P. regina* genome size using flow cytometry [35].

**Table 1:** Hi-C integrated *P. regina* genome assembly showed better quality than the genome published earlier.

Genome Assembly		
**Quality**	**Male P.regina genome** **(Andere et al. 2016)**	**Hi-C integrated Assembly** **(Male)**
No. of Contigs	1877	149
Contigs N50 (Megabase)	0.007 MB	7.01 MB
Total Length (Megabase)	534 MB	534 MB
Shortest Contig(base)	3	30,412
BUSCO Completeness (%)	96.77	99.3
**Genome Annotation**		
Gene Counts	9490	28,518
No. of Genes Hit BLAST	8789	10,693
No. of Exons	33,298	150,928

Metrics used to assess assembly quality include numbers of contigs, shortest contig length, number of exons, contig N50, gene counts and BUSCO completeness. The duplicates from the assembly were purged by HiFiasm and purge_dups. N50: median contig length. BUSCO: Benchmarking Universal Single-Copy Orthologs.

### The transition between feeding and wandering behavior during *P. regina* maggot development

**Fig 4:**
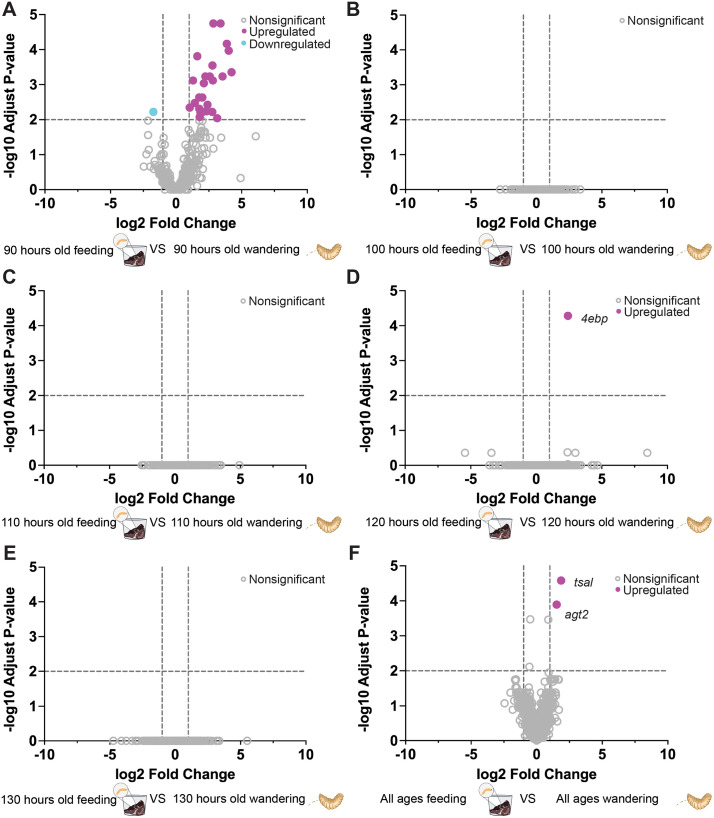
Age-dependent transcriptional changes associated with the transition from feeding to wandering in *P. regina* maggots. **(A–F)** Volcano plots showing differentially expressed genes (DEGs) between feeding and wandering larvae across different age cohorts: (A) 90 h, (B) 100 h, (C) 110 h, (D) 120 h, (E) 130 h, and (F) combined age groups. Differential expression was determined using DESeq2, with thresholds set at fold change > 2 (|log₂ fold change| > 1) and adjusted p-value < 0.01 (−log₁₀ padj > 2). In the volcano plot, the x-axis represents log₂ fold change, and the y-axis represents −log₁₀ adjusted p-value, with dashed lines indicating these thresholds. Genes are color-coded: upregulated (magenta), downregulated (cyan), and nonsignificant (gray). Notable genes such as *4ebp*, *agt2*, and *tsal* are labeled in panels D and **F.** Statistical significance was assessed using the Wald test. NS denotes genes without significant differences in expression (~10%).

**Fig 5:**
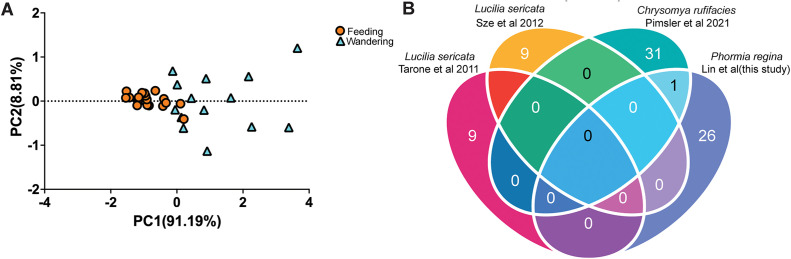
*cstB* is differentially expressed between the feeding and wandering stage maggots in both *Chrysomya rufifacies* and *Phormia regina.* (A) Principal component analysis (PCA) of gene expression profiles between feeding and wandering third instar maggots. PCA was performed using normalized gene expression data across all treatments to assess transcriptional differences between feeding (orange circles) and wandering (blue triangles) stages. PC1 and PC2 accounted for 91.19% and 8.81% of the total variance, respectively. (B) Venn diagram comparing the gene expression changes associated with feeding and wandering in third instar maggots from *Lucilia sericata* [24,25] and *Chrysomya rufifacies* [26], and *Phormia regina*. The numbers indicate the total number of differentially expressed genes reported in each study.

We identified 25 genes that displayed consistent upregulation and one downregulated gene when comparing gene expression in maggots age 90 h that are wandering to 90 h feeding maggots (Fig 4A). However, there were no differentially expressed genes when comparing feeding and wandering behaviors at other age cohorts except one upregulated gene at 120 h (Fig 4B-E). When comparing feeding to wandering across all age cohorts we did find two significant differentially expressed genes *tsal* and *agt2* (Fig 4F). The distinct transcriptomic shift at 90 h corresponds to the initiation of the wandering phase—a developmental transition characterized by maggots leaving carrion and searching for a place to pupate (Fig 1). The expression profiles indicated that most genes were downregulated during the wandering stage but upregulated during the feeding stages (S3A and S3B Fig). Principal component analysis (PCA) of normalized gene expression revealed a clear separation between feeding and wandering treatments along PC1, which accounted for 91.19% of the total variance (Fig 5A). Feeding samples formed a tight cluster, while wandering samples were more dispersed but generally grouped apart, indicating consistent transcriptomic differences between the two behavioral states. Our results suggest that most of the changes in gene expression between these states occur at its initiation.

To see if the genes associated with the transition from feeding to wandering behavior were conserved in other blowfly species, we compared differentially expressed genes in our dataset with previously published gene sets from *Lucilia sericata* [24,25] and *Chrysomya rufifacies* [26]. Surprisingly, there was little overlap in gene expression changes amongst the blowfly species characterized (Fig 5B). Only *cstB* (Cathepsin B) was associated with this transition in both *P. regina* and *C. rufifacies*.

### Dynamic gene expression profiles during blowfly maggot development revealed by DEGs and WGCNA

**Fig 6:**
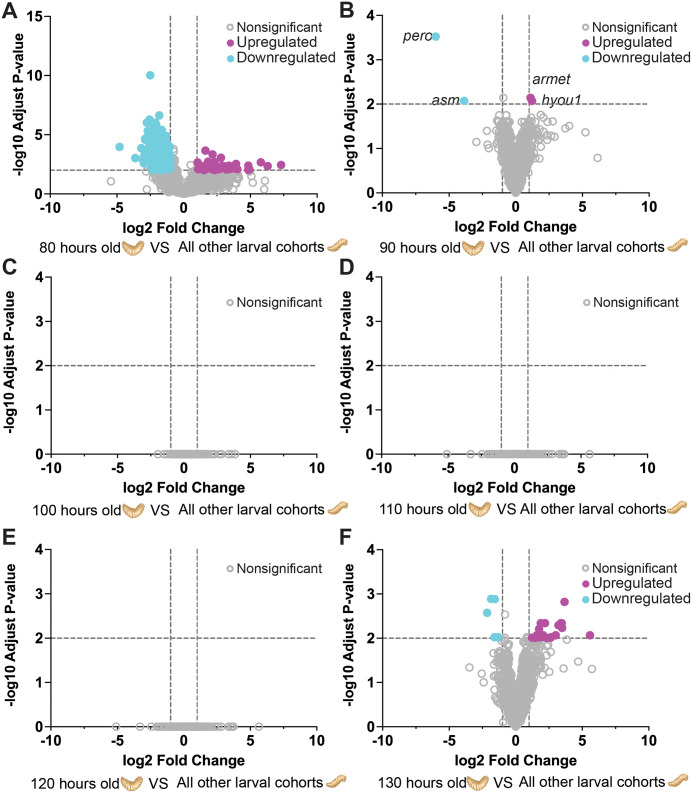
Age-specific gene expression signatures in *P. regina* maggots across developmental cohorts. **(A–F)** Volcano plots showing differentially expressed genes (DEGs) in each larval age group compared against all other age cohorts: (A) 80 h, (B) 90 h, (C) 100 h, (D) 110 h, (E) 120 h, and (F) 130 **h.** Differential expression was determined using DESeq2, with thresholds set at fold change > 2 (|log₂ fold change| > 1) and adjusted p-value < 0.01 (−log₁₀ padj > 2). In the volcano plot, the x-axis represents log₂ fold change and the y-axis represents −log₁₀ adjusted p-value, with dashed lines indicating these thresholds. Genes are color-coded as follows: upregulated (magenta), downregulated (cyan), and nonsignificant (gray). Notable differentially expressed genes such as *pero*, *asm*, *armet*, and *hyou1* are labeled in panel **B.** Statistical significance was assessed using the Wald test. NS denotes genes without significant differences in expression.

When comparing the gene expression profiles of two conditions at older larval cohorts (90–130 h) to the 80 h cohort, volcano plots revealed the presence of twenty differentially expressed genes (DEGs), with a noticeable preponderance of downregulated genes in older larval cohorts (**Fig 6A**). Expanding our investigation to a comparison from the other age groups (80 h, 100–130 h) to a 90 h cohort, volcano plots identified two upregulated and two downregulated DEGs (**Fig 6B**). In contrast, when we compared the gene expression profiles at 100 h, 110 h, and 120 h to other age groups, the analysis did not reveal any significant DEGs (Fig 6C-E). We conducted a comprehensive analysis of DEGs by comparing the earlier age cohorts (80 h-120 h) to a 130 h cohort. The resulting volcano plot showed a total of eighteen DEGs, the majority of which were upregulated at early larval cohorts (**Fig 6F**). Using a heatmap of DEGs, we compared other age cohorts against the 80 h maggots; the heatmap emphasizes that most DEGs were upregulated between 80–90 h but downregulated in older maggots (S4A-C Fig).

**Fig 7:**
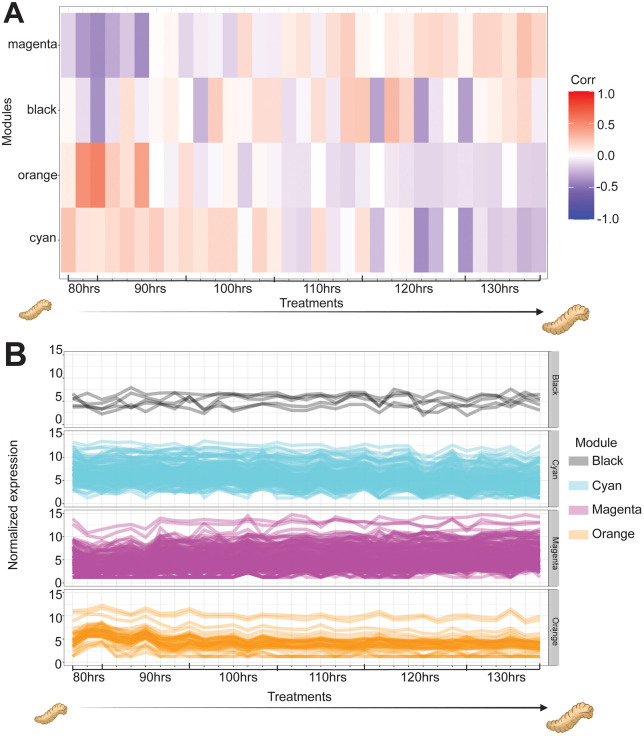
Weighted gene co-expression network analysis (WGCNA) reveals gene expression modules associated with age in maggots. (A,) Heatmaps of module–trait correlations showing representation of normalized reads of sample clusters (column dendrograms) by developmental age. Color scale indicates the strength of correlation between module eigengenes. (B) Expression profiles of genes within selected WGCNA modules (magenta, orange, black, cyan) across samples, arranged by age. These modules exhibit coordinated gene expression patterns associated with maggot age.

To ascertain the consistency of gene expression patterns across larval aging, we applied Weighted Gene Co-expression Network Analysis (WGCNA). The module–trait heatmap (**Fig 7A**) summarizes the association between each module eigengene (color-coded) and experimental traits (treatments). All genes associated with the color modules (traits) are provided in **S2 Table**. This analysis revealed a set of 168 genes characterized by co-expression patterns that either decreased or increased nearly synchronously over time. Examination of the magenta, orange, black and cyan modules highlighted distinct directional correlations in transcript expression across the developmental continuum (**Fig 7B**).

### GO enrichment analysis identifies molecular pathways associated with blowfly maggot development

To gain insight into the biological relevance of the candidate genes identified from DEG analysis, we conducted a statistical enrichment analysis of Gene Ontology (GO) terms. This analysis helped determine whether certain biological processes, cellular components, or molecular functions were overrepresented in each gene set compared to a reference set. We applied the DEG list from the age cohorts to GO enrichment, revealing substantial involvement of DEGs at older larval cohorts (90–130 h), compared to the 80 h age cohort, in processes related to ribonucleoprotein, small nuclear ribonucleoprotein assembly, and ribosome components (S5 Fig). Conversely, the DEGs at 90 hours were associated with processes related to signal transduction (S6 Fig). The DEGs at 130 hours pointed to significant involvement of peroxidase activity (S7 Fig). Regarding transition from feeding to wandering, we applied the gene list from the DEG analysis to GO enrichment (S8 Fig). The results suggested that redox metabolism might influence wandering behavior prior to pupation, potentially due to anaerobic glycolysis in midgut metabolism [36]. Additionally, laccase activity was found in the epicuticle of wandering *L. cuprina* maggots, indicating a role for phenol oxidation in cuticular sclerotization while they are about to pupariate [37, 38]. During the wandering stage, sterol transport and metabolism occur, likely contributing to the distribution of fat in adipose tissues for storage [39, 40].

### Nine genes associated with aging maggots are candidate molecular markers for improving forensic investigations

**Fig 8:**
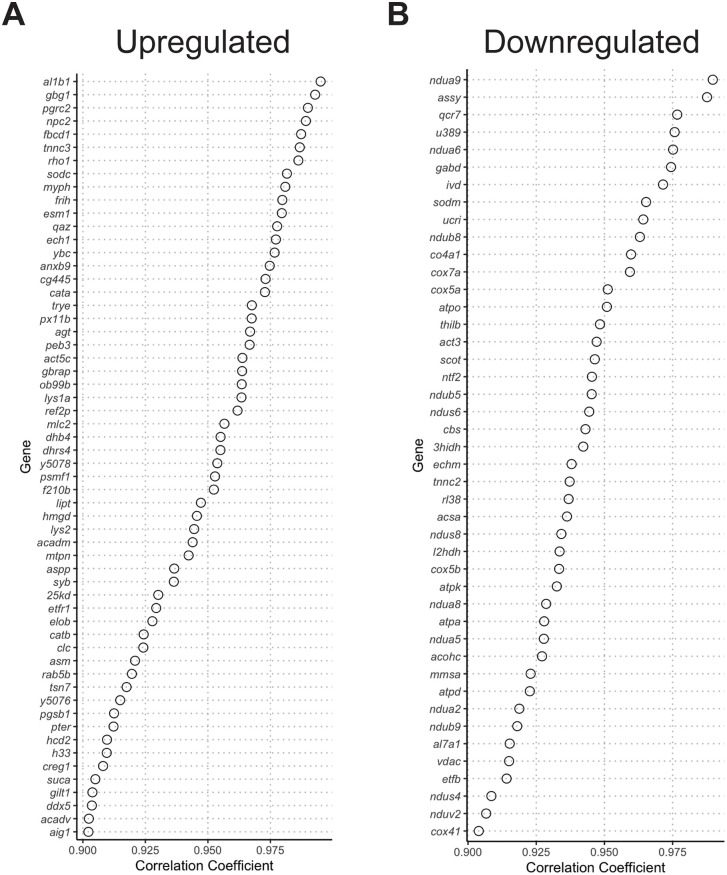
Age-associated candidate *P. regina* transcripts identified by linear regression analysis. Linear regression was used to identify transcripts with consistent changes in expression across developmental time points (80 h to 130 **h)**. A total of 59 transcripts were classified as upregulated **(A)**, showing increased expression over time with positive regression slopes (β₁ > 1) and strong model fit (*R²* ≥ 0.91). On the other hand, 45 transcripts were classified as downregulated **(B)**, exhibiting decreased expression with negative slopes (β₁ < –1) and *R²* ≥ 0.91. Gene lists are ranked by correlation coefficient, and expression is reported in transcripts per million (TPM).

**Fig 9:**
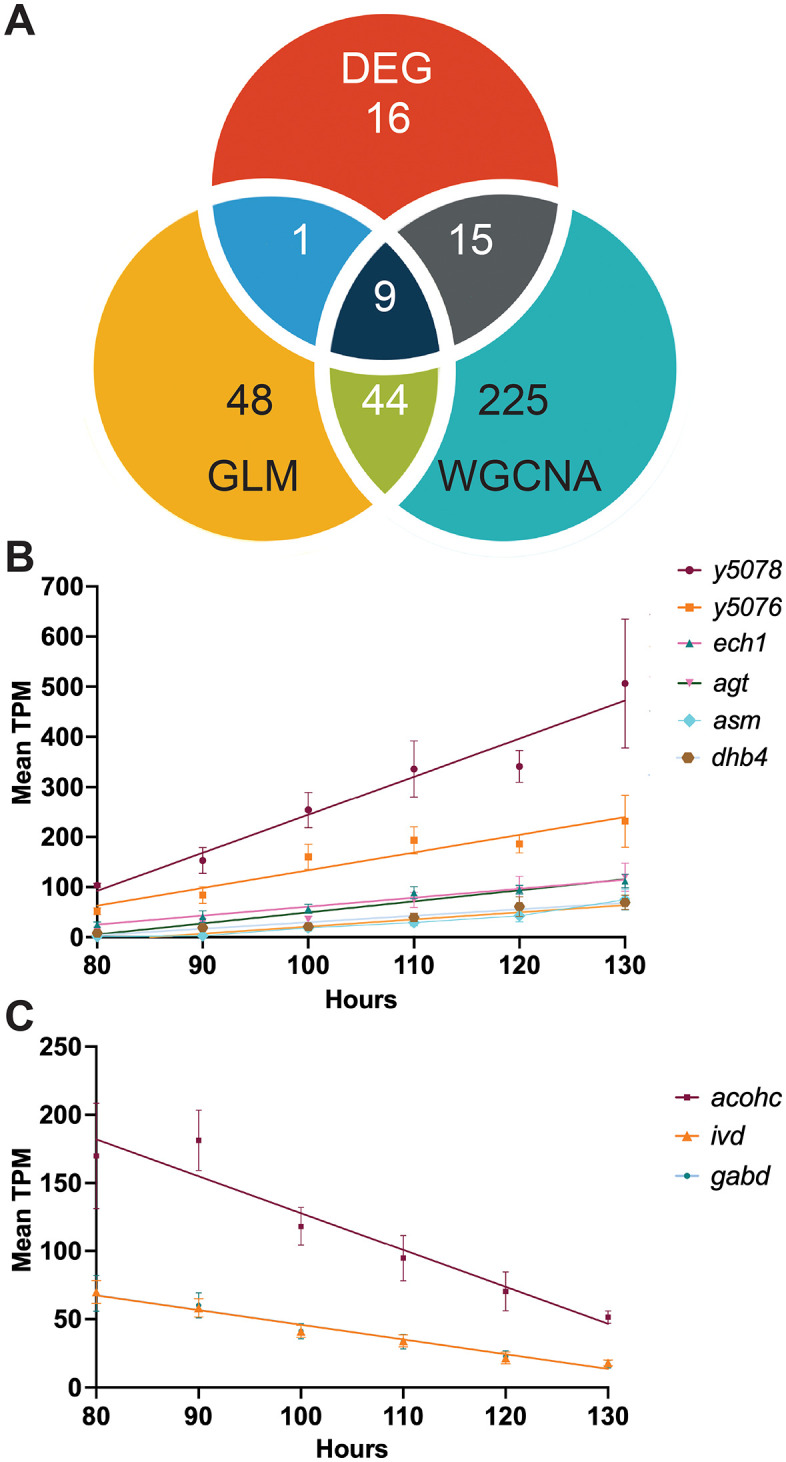
Nine genes were candidates for estimating maggot age across all three statistical analyses. **(A)** This Venn diagram displays a total of nine candidate genes that were consistently identified in all three analyses. GLM (General Linear Regression), WGCNA (Weighted Gene Co-Expression Network Analysis), and DE (Differentially Expressed Genes) analysis. The diagram illustrates the numbers of expressed transcripts associated with these three analytical approaches. Within those nine candidate genes, (B) six candidate genes (*y5078*, *y5076*, *agt, asm, ech1 dhb4*) appeared upregulated linear regression patterns. **(C)** Three candidate genes (*gabd, acohc, ivd) appeared* downregulated linear regression patterns.

General linear regression models (GLM) allow for the identification of useful markers that exhibit linear expression patterns over time. This is useful for accurate age determination of maggots in forensic investigations using methods such a quantitative or digital PCR. In our study, we applied the GLM to the normalized expression profiles of each gene over time, identifying a total of 58 upregulated genes and 44 downregulated genes that fit to this linear pattern (Fig 8). We also identified 10 genes, characterized by unvarying TPM values, that may be useful as controls in quantitative PCR (S9 Fig). We considered the most promising candidate genes to be those identified by all of our three analyses: DEG, GLM, and WGCNA (Fig 9A). This approach revealed nine genes. Among these genes, *y5078*, *y5076*, *agt2, ech1, dhb4, asm* were upregulated; while *gabd, acohc, ivd* were downregulated (Fig 9B and 9C).

## Discussion

Despite the practical and basic scientific value of the Calliphoridae, there has been relatively little genomic investigation of the group [41]. The high-quality, chromosomal-level genome assembly presented here represents a significant improvement over previous assemblies of blowflies [34]. This will help open up these forensically important insects to further genomic and genetic analyses. We used our updated genome to provide comprehensive gene models for our transcriptome analyses of maggot aging [42]. We identified 41 transcripts by DEG, 293 transcripts by WGCA, and 102 transcripts by GLM analyses. These genes are candidates that are likely to improve age estimation of *P. regina* larvae. Out of these 358 transcripts, we highlight nine transcripts — *y5078, y5076, agt2*, *ech1*, *dhb4*, *asm*, *acohc, gabd* and *ivd* — that were supported by all three methods of analysis as our most promising candidates for the molecular estimation of maggot age.

We show that *y5078* (Y5078_DROME uncharacterized protein from *Drosophila melanogaster*), *y5076* (Y5076_DROME uncharacterized protein from *Drosophila melanogaster*), *agt2* (alanine glyoxylate aminotransferase), *ech1* (dienoyl-coenzyme A isomerase), *dhb4* (peroxisomal multifunctional enzyme type 2) and *asm* (sphingomyelin phosphodiesterase) are upregulated as maggots age. The genes *acohc* (cytoplasmic aconitate hydratase), *gabd* (succinate-semialdehyde dehydrogenase) and *ivd* (isovaleryl-CoA dehydrogenase) are downregulated as maggots age. These transcripts clearly delineate the aging of maggots from 80 h to 130 h making them suitable markers for age estimation. In addition, our results provide new insights into the regulatory network underpinning homeostasis and metabolism during third instar maggot development. For example, we observed the upregulation of *ech1* (dienoyl-coenzyme A isomerase) and *dhb4* (peroxisomal multifunctional enzyme type 2), which are significant components of the peroxisomal pathway of lipid metabolism, particularly the α-oxidation process in *D. melanogaster* [43, 44]. This upregulation suggests that *ech1* and *dhb4* are pivotal for lipid metabolism, a critical factor for larvae to complete their development and transition into pupae and ultimately adults. The elevated expression of *asm* (sphingomyelin phosphodiesterase) genes in larvae hints at events related to cellular development and differentiation, specifically in the context of sphingolipid metabolism [45]. On the other hand, the downregulation of *acohc* (cytoplasmic aconitate hydratase) and *gabd* (succinate-semialdehyde dehydrogenase) during development implies their potential roles in translational control of proteins, possibly in response to iron regulation as larvae progress into pupal stages [46, 47]. Moreover, downregulated of *ivd* (isovaleryl-coA dehydrogenase) expression suggested a potential developmental shift away from amino acid oxidation to alternative energy metabolism [48]. The upregulated marker genes have been associated with cholesterol export, energy metabolism, fatty acid metabolism, and detoxification [49–53]. On the other hand, downregulated marker genes participate in regulation of iron homeostasis and GABA degradation pathway as maggots age [54, 55].

Our study identified 27 transcripts from DEGs associated with feeding and wandering behavior. Notably, the presence of DEGs at 90 hours, consistent with the emergence of wandering maggots at 90 hours, suggests a strong connection between these genes and the behavioral transition. Two genes, *agt2* (alanine glyoxylate aminotransferase) and *tsal* (L-threonine ammonia-lyase) across all age cohorts indicating a potential role for amino acid homeostasis during transition from feeding to wandering. [56, 57]. By comparing genes associated with feeding and wandering stages from other studies (**Fig 5B**), we found *cstB* (cathepsin B) to be the only overlapping gene between any blowfly species. Expression of *cstB* has been associated with tissue remodeling and fat degradation during the transition from larva to pupa [58,59], consistent with its potential conserved role across blowflies. Interestingly, most genes identified in our analysis did not overlap with those reported in *L. sericata* [24,25], and *C. rufifacies* [26]. Some factors may contribute to this lack of overlap. First, differences in methodology can make it challenging to compare the gene datasets. Second, developmental transitions may be regulated by species-specific gene networks, such that the same functional pathways are engaged but through different sets of genes. This interpretation is supported by the gene ontology-level similarities reported both in *L. sericata* [25], *C. rufifacies* [26] and *P. regina*, which highlights conserved categories such as peroxisome organization and micromolecule modification. Finally, limited sample sizes in studies may constrain the detection of overlapping genes across datasets.

Previous study has shown that miRNAs (e.g. miR-92b) are detectable in larvae during the feeding stage and in the early wandering phase, but are absent in late wandering larvae [23]. This suggests that the presence or absence of food-derived RNAs could serve as an additional molecular marker for distinguishing between these stages. While our study focused on host gene expression, the dynamics of small RNA associated with food consumption may contribute to the transcriptomic signatures we observed, particularly in separating feeding from wandering larvae. Incorporating feeding-associated small RNAs into forensic age estimation frameworks may therefore improve precisions, especially in cases where larvae are collected at or near the wandering stage.

While maggot size or weight are often used as an age indicator, they remain imperfect metrics due to substantial variation at a given age, particularly during later larval development [2,60–62]. Similarly, as seen in these results the age of transition from feeding to wandering is also highly variable, making that milestone of limited use for estimating age (**Fig 1A**). By combining enhanced transcriptional information, our study identifies candidate genes that not only contribute to a better understanding of dipteran development and behavior during the third larval instar stage but also have potential applications in forensic investigations for estimating time since death. Field studies are needed to validate the performance of these candidate genes in *P. regina* and to facilitate the development of genetic marker kits that can ultimately improve precision in time-of-death estimation.

## Materials and methods

### Identifying the transition between feeding and wandering behavior

We maintained a laboratory colony of an inbred line of *Phormia regina* originally from Lincoln, Nebraska [63]. Developmental rate data were available [63]. All rearing containers were maintained at 27.5 ± 0.5 °C. under 16:8 light:dark cycle in one SMY04–1 DigiTherm CirKinetics Incubator (TriTech Research, Inc., Los Angeles, CA, USA). The incubator was equipped with uniform lighting, additional fans, and a port for thermometer access.

Each equal-age cohort was reared within a plastic 72 x72x100 mm insect breeding box (#310075, SPL Life Sciences, Miramar, FL, USA). Each box contained 0.5 cm sawdust and a 30g of fresh chicken liver in a suspended 4 oz paper cup (S1 Fig). Eggs were obtained on a paper towel soaked with chicken liver blood placed in a cage of *P. regina* adults for 30 minutes before removal. The removal time was considered age zero for those insects. About 500–1000 newly deposited eggs were placed on wet paper in a covered petri dish. The eggs were kept at high humidity over an open water container at 27.5 ± 0.5 °C. After 24 hours, fifteen newly hatched maggots were transferred into each aliquot of fresh liver inside the rearing box. All boxes were reared at the same 27.5 ± 0.5 °C incubator. For each of four trials, 28 boxes with maggots were set up. During each sampling time, four rearing boxes were randomly removed from the incubators. We distinguished a maggot as feeding if it was still on the liver and wandering if it was in the sawdust. Sampling involved removal of an entire cohort at a preselected age. Sampled maggot was individually placed in a 1.5mL microcentrifuge tube with 1mL of Thermofisher RNAlater (Invitrogen, MA, USA) at 4 °C. Each tube was labeled with numbers for RNA analysis. After 24 hours, the RNAlater was removed from the tube and the insect was stored at -80 °C.

### RNA & DNA sequencing

Over three independent rearing cohorts, individual maggots were selected based on their age, behavior, and average weight relative to other members of the same cohort. Maggots exhibiting feeding behavior (F) or wandering behavior (W) were collected at 10-hour intervals from 80 to 130 hours post-hatching. The specimens were homogenized using a pestle with 200 μl of ambion TRIzol reagent (MA, USA). RNA was extracted from using a QIAGEN RNeasy Plus extraction kit (MA, USA). The concentration of the RNA samples was assessed with an Invitrogen Qubit Fluorometer using a Qubit RNA HS Assay kit (MA, USA). The integrity of the extracted RNA was evaluated with a Bioanalyzer using an Agilent RNA 6000 Pico chip. A total of 33 RNA samples from maggots of known ages were sent to BGI (CA, USA) for 150 bp paired-end RNA sequencing, and 2 RNA samples from adult flies following the same RNA extraction as previously described were sent to Genewiz for 150 bp paired-end RNA sequencing (NJ, USA).

A male virgin adult blowfly was sent to PacBio for sequencing using the PacBio Sequel II platform to generate a HiFi read library. Additionally, another male virgin adult blowfly was sent to CD Genomics, where Hi-C libraries were quantified and sequenced on the Illumina NovaSeq platform (San Diego, CA, USA). See S1 Table for details on short-read and long-read libraries.

### Iso-seq mRNA analysis of *P. regina*

A single virgin adult male, a single virgin adult female, a 110 h feeding third instar maggot, and a 110 h wandering maggot were sent to DNA Sequencing Center at Brigham Young University for Iso-seq. Iso-Sequencing was performed on the PacBio Sequel II platform. Consensus sequences were generated from the SMRTBell libraries and collapsed into isoforms by Isoseq v3.0. The long reads libraries were used as evidence for genome annotation.

### Building a new *P. regina* genome assembly

We performed *de novo* assembly using HiFiasm v0.16.1 on a HiFi long-read library from a single adult male blowfly (See **RNA & DNA sequencing**), adopting purge mode to generate both haplotype and diplotype assemblies of *Phormia regina* [64]. Duplicate contigs were further eliminated by purge_dups v1.2.6 [65]. The contaminated contigs were identified by NCBI BLAST against a publicly available nucleotide database and filtered by Blobtool v2.6.4 [66]. The repeat element boundaries and repeat database was *de novo* assembly by the clean contigs using RepeatModeler v2.0.2 [67–75]. Hi-C sequencing libraries from a male adult blowfly (See **RNA & DNA sequencing**), were first mapped to the clean contigs by Arima mapping pipeline and the.bam file was used to perform chromosomal integrated assembly by yahs v1.1 [76]. A Hi-C heat map was generated by juicebox v2.20.00 [77]. The curation and chromosomal boundaries were manually edited following the Genome Assembly Cookbook [78]. The chromosomal genome was annotated using the Funannotate v1.8.13 annotation pipeline, with Iso-seq libraries providing support for evidence-based gene prediction during the annotation process. (See **Iso-seq mRNA analysis of *P. regina***) [79–84].

### Gene expression analysis

The quality of RNA seq libraries previously described (See **RNA & DNA sequencing**) were initially assessed by FastQC v0.11.9 [85]. The adapter sequences from the short reads were trimmed by Trimmomatic v0.39 [86]. The clean short read libraries were mapped to the newly annotated genome (See **Building a new *P. regina* genome assembly section**) using Salmon v1.8.0 for quantification [87]. Count matrices were obtained by tximport 1.34.0 [88]. Differential expression analysis was performed by DeSeq2 1.34.0 Bioconductor [89]. The gene names were manually curated based on the results from the Blastn against orthologous UniProt v2023_02. Pairwise comparison was performed among all treatments to identify differentially expressed transcripts (> 2-fold change, *a* < 0.01 FDR) using DEBrowser V3.18 [90]. Gene co-expression *a*nalysis was produced by DESeq2 v1.34.0 followed by tidyverse v2.0.0, magrittr v2.0.3, and WGCNA v1.72.1 analysis on RStudio v0.15.0 (R version 4.1.2) [91, 92]. Gene ontology enrichment analysis was performed by using ShinyGO v0.76 under FDR-corrected cut off *a *< 0.05 [92]. Princip*a*l component analysis (PCA) of normalized expression data was conducted in DEBrowser v3.18 [90].

### Comparison of gene expression datasets from other studies

Gene expression changes associated with feeding and wandering stages were obtained from published datasets on *Lucilia sericata* [24,25] and *Chrysomya rufifacies* [26]. For *L. sericata* [25], we extracted *Drosophila melanogaster* orthologs from expression clusters that exhibited stage-specific patterns during larval development. These clusters were based on transcript nodes with correlated RPKM values, grouped using hierarchical clustering. Using clusters enriched during feeding and wandering stages, we cross-referenced these published gene sets with differentially expressed genes identified in our *P. regina* dataset. For *C. rufifacies* [26], we compiled *D. melanogaster* orthologs from cluster 22 as well as candidate genes validated by qPCR that were reported to differ between feeding and wandering larvae. These gene lists were then cross-referenced with the *Phormia regina* differentially expressed genes identified in our study. We relied on what previous authors classified as changes in gene expression in their respective organisms and then compared those datasets with the results from our current study. Overlaps among gene lists were analyzed using InteractiVenn [93]. The four circles Venn diagram was drawn using Adobe Illustrator V29.8.1.

### Finding candidate genes for predicting larval age

Raw reads from the development of aging maggot as previously described were normalized to TPM values by tximport v1.34.0 [94]. Linear regression model was applied to define the housekeeping genes (*s* < -2, all values greater than 0), upregulated genes (β₁ > 1 and *R*^*2*^: 0.9 ~ 1) and downregulated genes (β₁ < -1 and *R*^*2*^: 0.9 ~ 1) from the mean TPM values across each age cohort; mean TPM values from the linear regression model were calculated over six replicates (including three feeding replicates and three wandering replicates) for age cohorts from 90 to 130 hours, and the 80-hour time point includes only three feeding replicates (See S1 Table). The correlation plots were created using ggplot2 [95]. The Venn diagram list was created by the VennDiagram v1.7.3 package from RStudio v0.15.0 (R version 4.1.2) and drawn by Procreate v5.3.7 from Savage Interactive Pty Ltd [96]. Linear regression figures were created by Prism10 v0.2.2 from GraphPad software. All the data used to generate the figures are available via the dryad data repository [97].

## Supporting information

S1 Table Metadata of RNA-seq and Iso-Seq libraries. Tabular metadata describing the biological and technical details of the RNA-seq and Iso-Seq libraries used in this study. Each row corresponds to a biological replicate used for transcriptomic or genomic analysis. Variables include sample name, BioProject accession, organism, isolate, breed, isolation source, collection date, geographic origin, tissue type, age, weight, explanatory notes (feeding vs. wandering), and file names for sequencing reads deposited in NCBI SRA (PRJNA990781).
(XLSX)

S2 Table WGCNA gene Dicership statistics. Per gene module membership statistics from the weighted gene co-expression network analysis (WGCNA). Each row corresponds to a gene that passed the variance filter and was included in colored modules. Variables include gene ID, assigned module, module membership correlation (kME) with eigengene, and associated two-sided p-value.
(CSV)

S1 Fig The larval rearing box used in the study disassembled (A) and assembled (B). A paper cup containing 30g chicken liver was suspended over a layer of sawdust with a loop held by a magnet. The boxes were placed in a 27.5°C incubator. Wandering maggots would leave the cup and fall into the wood shavings. Maggots were visually scored for feeding (in paper cup) or wandering behaviors (in the sawdust) and weighed.
(TIF)

S2 Fig Median weight of wandering maggots is significantly higher than the median weight of feeding maggots. The figure presents findings of comparing the weights of feeding and wandering maggots across ten age cohorts spanning from 80 to 130 h. Maggots were categorized into feeding or wandering groups based on their behavior. The median weight of wandering maggots significantly exceeded that of feeding maggots according to the Mann-Whitney test (*p *< 0.0001). Each maggot’s weight is depicted by a dot, with the center horizontal line of each box indicating the median weight. Additionally, error bars depict the standard error.
(TIF)

S3 Fig *agt2* and *tsal* were differentially expressed genes in feeding and wandering behavior during larval development across all ages. The heat map showed differentiated expressed transcripts over aging maggots sorted into feeding (F) and wandering (W) at 90 h (A) and all the ages (B) behaviors from 80 h to 130 h. The figure showed the clusters of relationships (y axis) among the transcripts to the left and treatment on the x axis (numbers: age from 80 to 130 h). The heat map was generated by DESEq2 based on the Spearman correlation (fold change > 2, FDR < 0.01).
(TIF)

S4 Fig Heatmaps reveal distinct expression patterns of transcripts when comparing early and older aging cohorts. Three pairwise comparisons were conducted: (A) 80 h versus other aging cohorts, (B) 90 h old versus other aging cohorts, and (C) 130 h versus other aging cohorts, resulting in heat maps displaying distinct expression patterns of transcripts between early and older aging cohorts. Clusters of relationships among transcripts are presented on the right, with the treatment depicted on the x-axis (feeding (F), wandering (W), numbers: age from 80 to 130 h). The heat map, generated by DESEq2 using Spearman correlation, focuses on transcripts with fold change >2 and FDR < 0.01.
(TIF)

S5 Fig Gene Ontology (GO) enrichment analysis from 80 h maggots. This analysis highlights the most significantly enriched GO terms among genes differentially expressed at this developmental stage. Each bubble represents a GO term, categorized based on Biological Process, Cellular Component, or Molecular Function. The Gene Ratio represents the proportion of DEGs associated with a specific GO term relative to the total number of input DEGs. The Counts indicate the absolute number of DEGs mapped to each GO term in the database. Bubble size corresponds to the number of DEGs (Counts, indicating the absolute number of DEGs mapped to each GO term in the database), while color intensity reflects the statistical significance (adjusted p-value) of enrichment. Enrichment significance was determined using the Benjamini-Hochberg procedure with a false discovery rate (FDR) threshold of < 0.05. The Gene Ratio represents the proportion of DEGs associated with a specific GO term relative to the total number of input DEGs.
(TIF)

S6 Fig Gene Ontology (GO) enrichment analysis from 90 h maggots. This analysis highlights the most significantly enriched GO terms among genes differentially expressed at this developmental stage. Each bubble represents a GO term, categorized based on Biological Process, Cellular Component, or Molecular Function. The Gene Ratio represents the proportion of DEGs associated with a specific GO term relative to the total number of input DEGs. The Counts indicate the absolute number of DEGs mapped to each GO term in the database. Bubble size corresponds to the number of DEGs (Counts, indicating the absolute number of DEGs mapped to each GO term in the database.), while color intensity reflects the statistical significance (adjusted p-value) of enrichment. Enrichment significance was determined using the Benjamini-Hochberg procedure with a false discovery rate (FDR) threshold of < 0.05. The Gene Ratio represents the proportion of DEGs associated with a specific GO term relative to the total number of input DEGs.
(TIF)

S7 Fig Gene Ontology (GO) enrichment analysis from 130 h maggots. This analysis highlights the most significantly enriched GO terms among genes differentially expressed at this developmental stage. Each bubble represents a GO term, categorized based on Biological Process, Cellular Component, or Molecular Function. The Gene Ratio represents the proportion of DEGs associated with a specific GO term relative to the total number of input DEGs. The Counts indicate the absolute number of DEGs mapped to each GO term in the database. Bubble size corresponds to the number of DEGs (Counts, indicating the absolute number of DEGs mapped to each GO term in the database.), while color intensity reflects the statistical significance (adjusted p-value) of enrichment. Enrichment significance was determined using the Benjamini-Hochberg procedure with a false discovery rate (FDR) threshold of < 0.05. The Gene Ratio represents the proportion of DEGs associated with a specific GO term relative to the total number of input DEGs.
(TIF)

S8 Fig Gene Ontology (GO) enrichment analysis comparing feeding to wandering maggots. This analysis highlights the most significantly enriched GO terms among genes differentially expressed between feeding and wandering behaviors. Each bubble represents a GO term, categorized based on Biological Process, Cellular Component, or Molecular Function. The Gene Ratio represents the proportion of DEGs associated with a specific GO term relative to the total number of input DEGs. The Counts indicate the absolute number of DEGs mapped to each GO term in the database. Bubble size corresponds to the number of DEGs (Counts, indicating the absolute number of DEGs mapped to each GO term in the database.), while color intensity reflects the statistical significance (adjusted p-value) of enrichment. Enrichment significance was determined using the Benjamini-Hochberg procedure with a false discovery rate (FDR) threshold of < 0.05. The Gene Ratio represents the proportion of DEGs associated with a specific GO term relative to the total number of input DEGs.
(TIF)

S9 Fig Related to Fig 9. Linear regression analysis showed the genes whose expression did not vary over developmental time. Linear regression model showed 10 housekeeping genes expressed over time (80 h ~ 130 h) at transcripts per millions (TPM) values (*s* < -2, all values greater than 0).
(TIFF)
