# Supplementary figures and images for: Unraveling forensic timelines using molecular markers in Phormia regina maggots
Source: PLoS Genet. 2025 Dec 4;21(12):e1011948. doi: 10.1371/journal.pgen.1011948 (PMC12677486; doi:10.1371/journal.pgen.1011948)

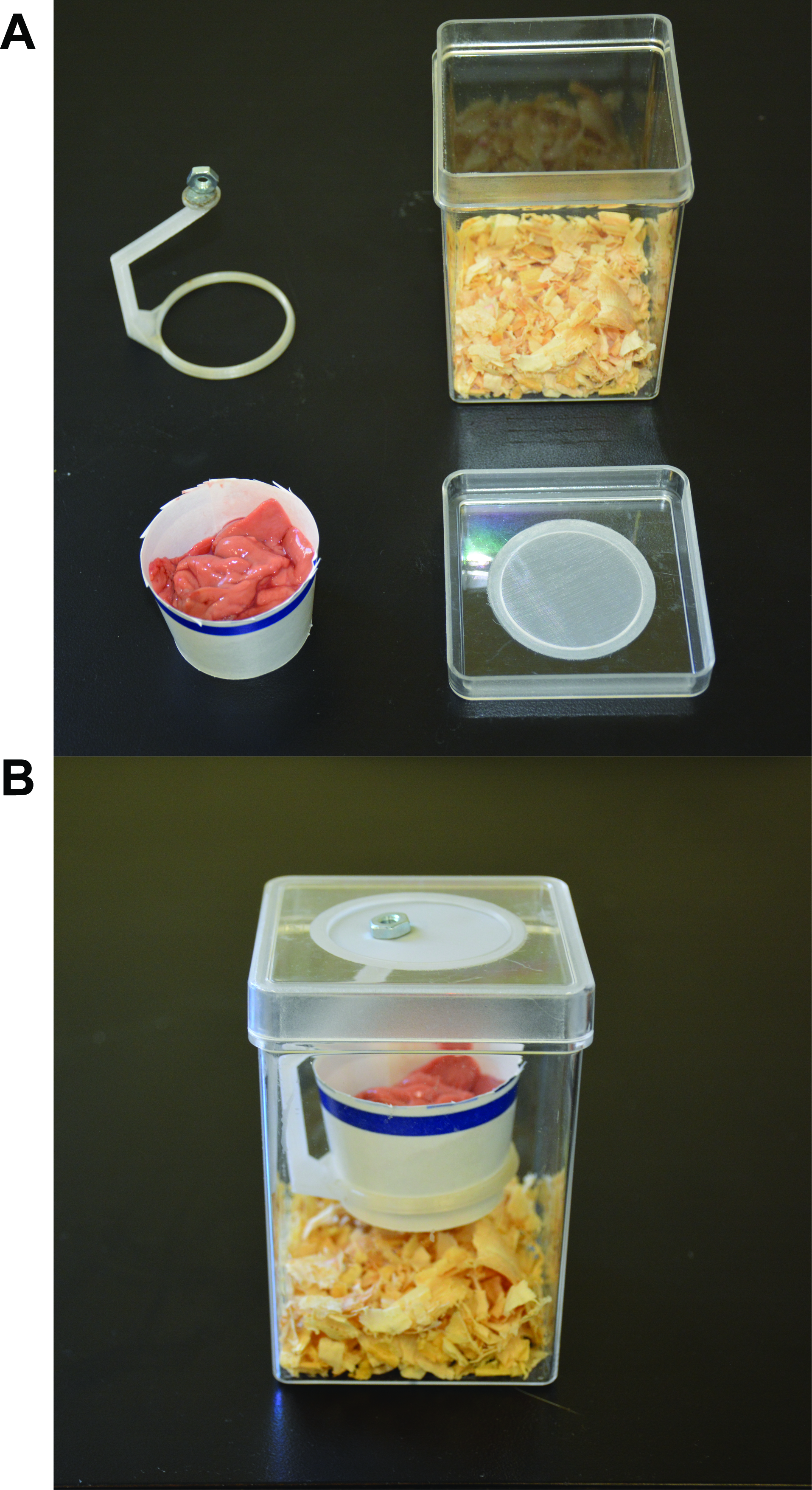

Supplement: S1 Fig — A paper cup containing 30g chicken liver was suspended over a layer of sawdust with a loop held by a magnet. The boxes were placed in a 27.5°C incubator. Wandering maggots would leave the cup and fall into the wood shavings. Maggots were visually scored for feeding (in paper cup) or wandering behaviors (in the sawdust) and weighed. (TIF) [file pgen.1011948.s003.tif]

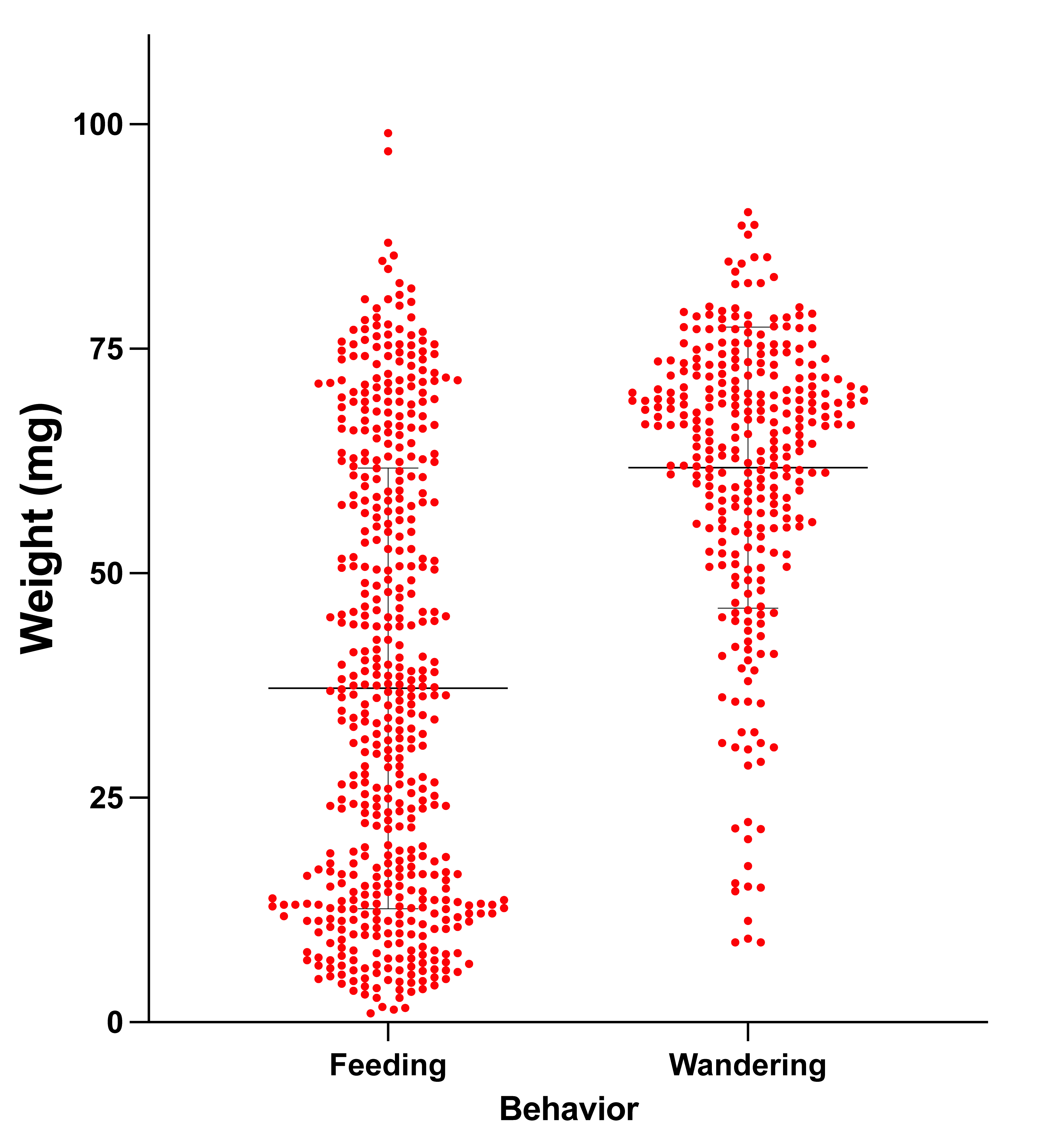

Supplement: S2 Fig — The figure presents findings of comparing the weights of feeding and wandering maggots across ten age cohorts spanning from 80 to 130 h. Maggots were categorized into feeding or wandering groups based on their behavior. The median weight of wandering maggots significantly exceeded that of feeding maggots according to the Mann-Whitney test (p < 0.0001). Each maggot’s weight is depicted by a dot, with the center horizontal line of each box indicating the median weight. Additionally, error bars depict the standard error. (TIF) [file pgen.1011948.s004.tif]

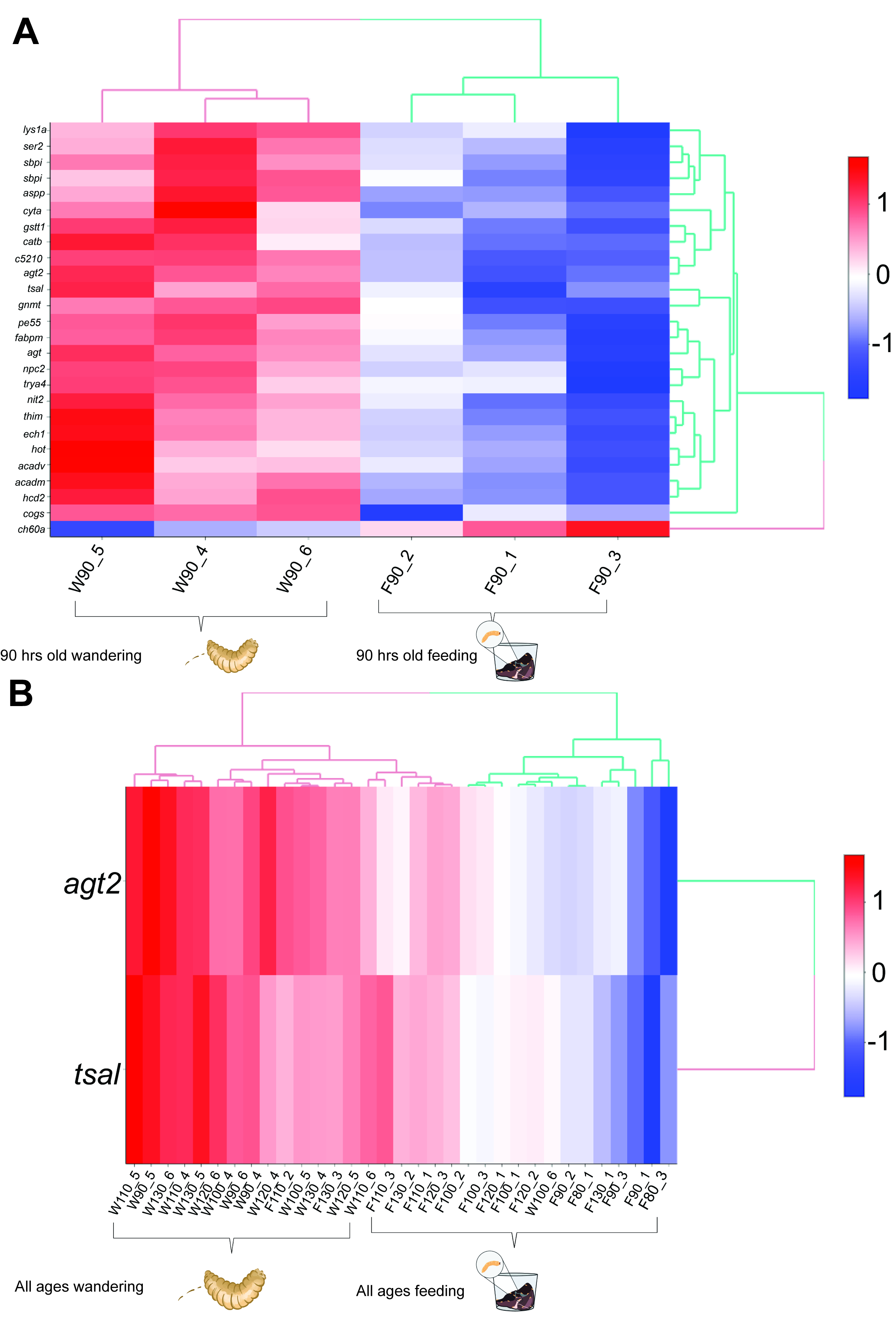

Supplement: S3 Fig — The heat map showed differentiated expressed transcripts over aging maggots sorted into feeding (F) and wandering (W) at 90 h (A) and all the ages (B) behaviors from 80 h to 130 h. The figure showed the clusters of relationships (y axis) among the transcripts to the left and treatment on the x axis (numbers: age from 80 to 130 h). The heat map was generated by DESEq2 based on the Spearman correlation (fold change > 2, FDR < 0.01). (TIF) [file pgen.1011948.s005.tif]

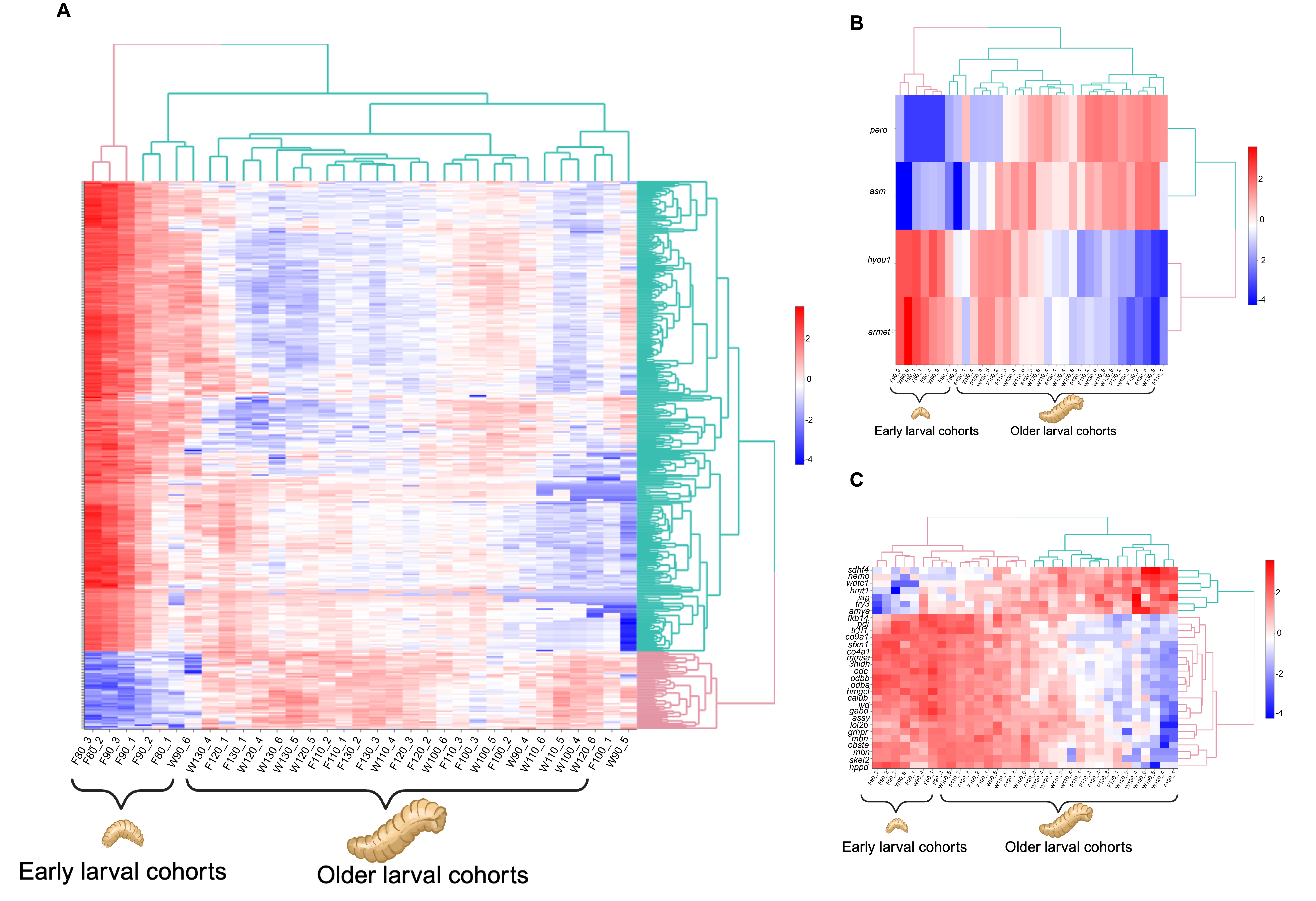

Supplement: S4 Fig — Three pairwise comparisons were conducted: (A) 80 h versus other aging cohorts, (B) 90 h old versus other aging cohorts, and (C) 130 h versus other aging cohorts, resulting in heat maps displaying distinct expression patterns of transcripts between early and older aging cohorts. Clusters of relationships among transcripts are presented on the right, with the treatment depicted on the x-axis (feeding (F), wandering (W), numbers: age from 80 to 130 h). The heat map, generated by DESEq2 using Spearman correlation, focuses on transcripts with fold change >2 and FDR < 0.01. (TIF) [file pgen.1011948.s006.tif]

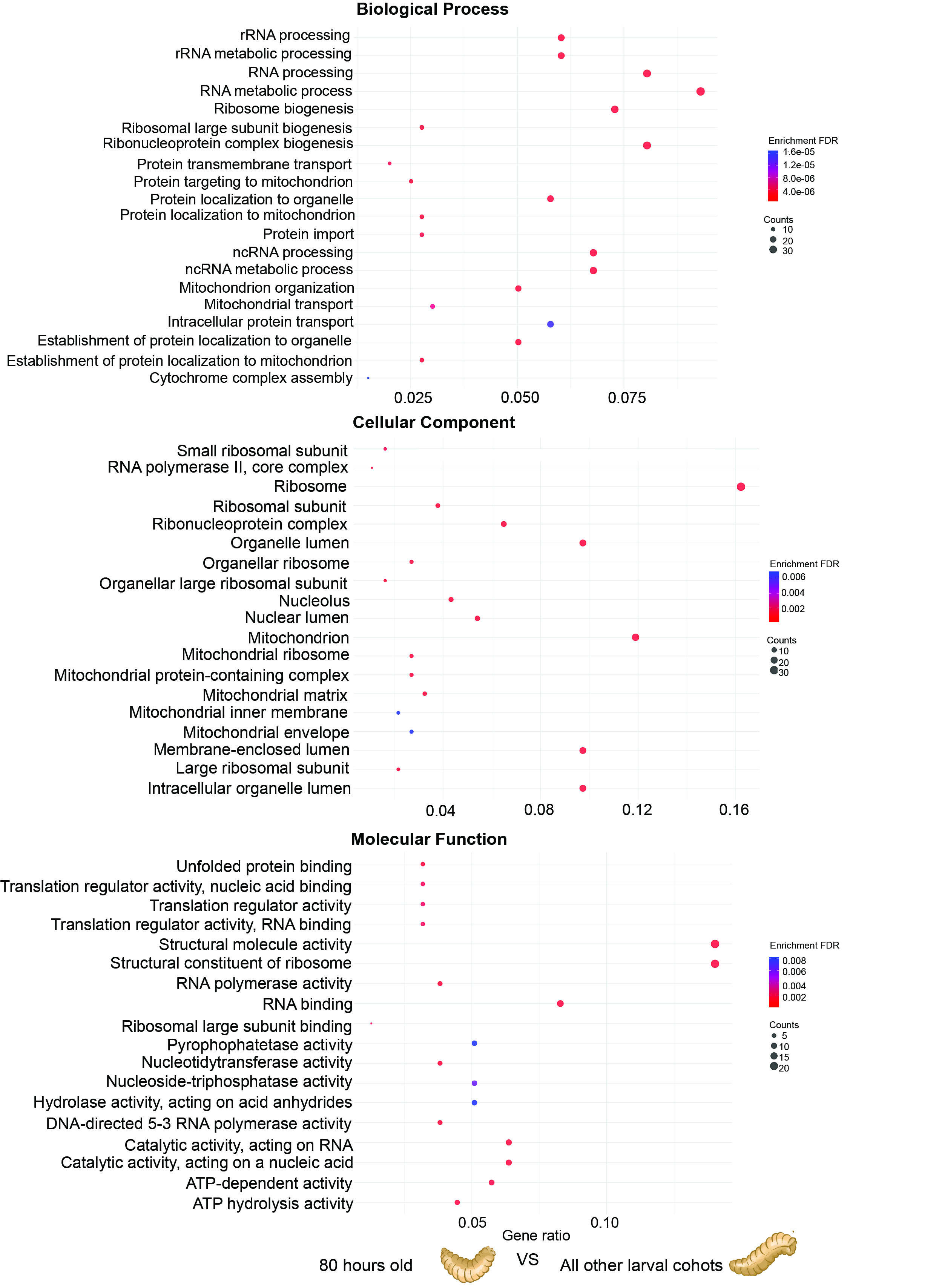

Supplement: S5 Fig — This analysis highlights the most significantly enriched GO terms among genes differentially expressed at this developmental stage. Each bubble represents a GO term, categorized based on Biological Process, Cellular Component, or Molecular Function. The Gene Ratio represents the proportion of DEGs associated with a specific GO term relative to the total number of input DEGs. The Counts indicate the absolute number of DEGs mapped to each GO term in the database. Bubble size corresponds to the number of DEGs (Counts, indicating the absolute number of DEGs mapped to each GO term in the database), while color intensity reflects the statistical significance (adjusted p-value) of enrichment. Enrichment significance was determined using the Benjamini-Hochberg procedure with a false discovery rate (FDR) threshold of < 0.05. The Gene Ratio represents the proportion of DEGs associated with a specific GO term relative to the total number of input DEGs. (TIF) [file pgen.1011948.s007.tif]

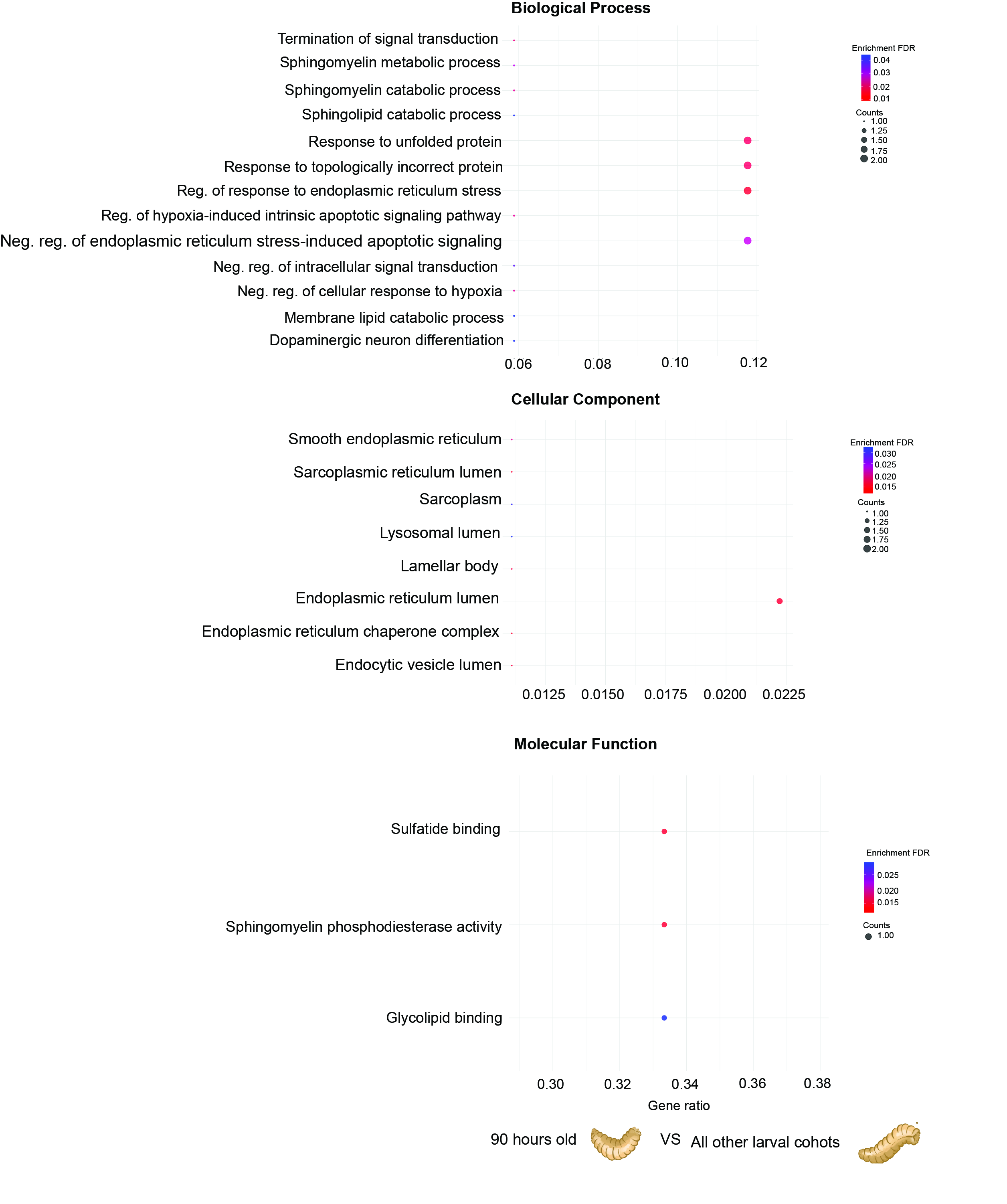

Supplement: S6 Fig — This analysis highlights the most significantly enriched GO terms among genes differentially expressed at this developmental stage. Each bubble represents a GO term, categorized based on Biological Process, Cellular Component, or Molecular Function. The Gene Ratio represents the proportion of DEGs associated with a specific GO term relative to the total number of input DEGs. The Counts indicate the absolute number of DEGs mapped to each GO term in the database. Bubble size corresponds to the number of DEGs (Counts, indicating the absolute number of DEGs mapped to each GO term in the database.), while color intensity reflects the statistical significance (adjusted p-value) of enrichment. Enrichment significance was determined using the Benjamini-Hochberg procedure with a false discovery rate (FDR) threshold of < 0.05. The Gene Ratio represents the proportion of DEGs associated with a specific GO term relative to the total number of input DEGs. (TIF) [file pgen.1011948.s008.tif]

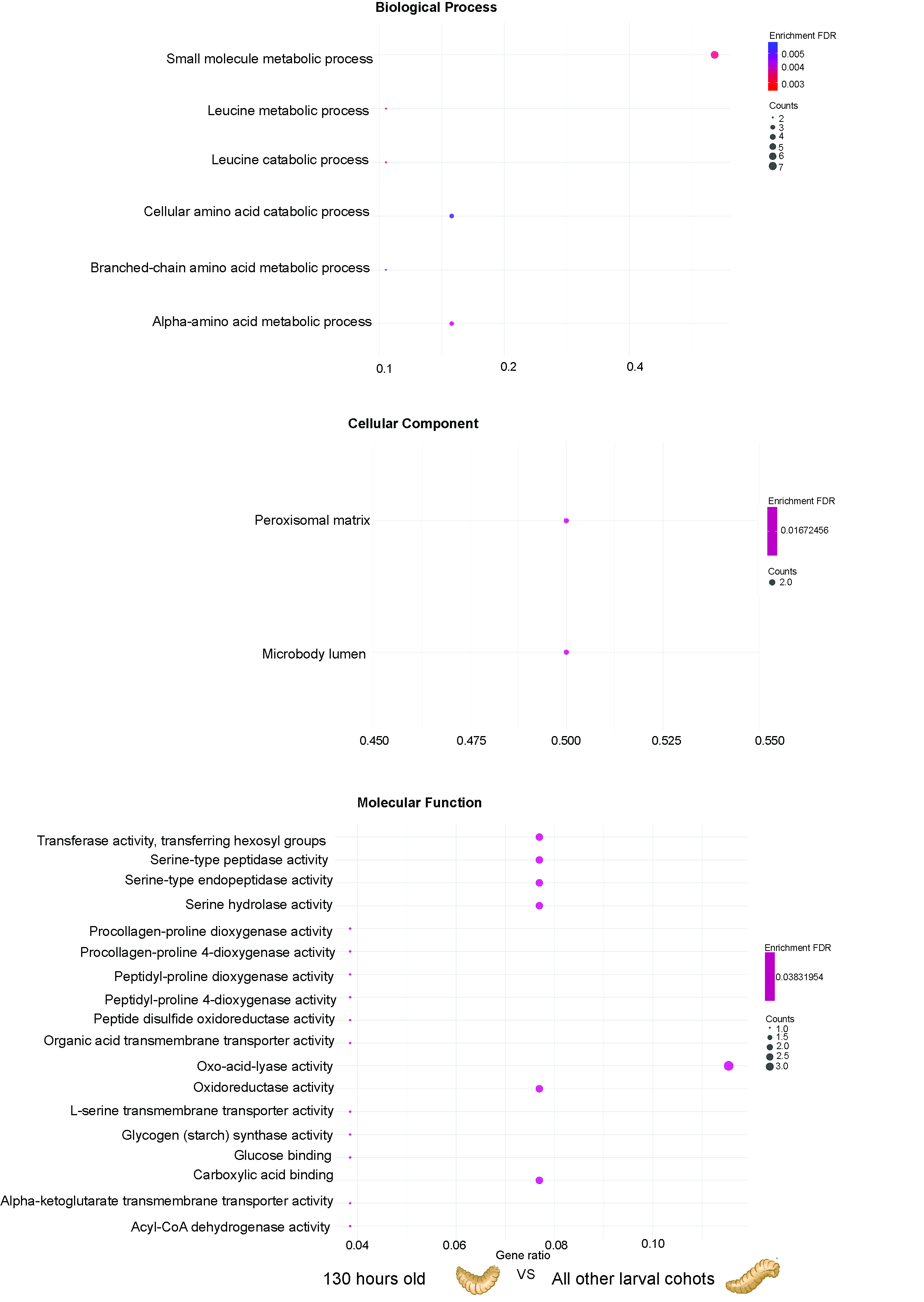

Supplement: S7 Fig — This analysis highlights the most significantly enriched GO terms among genes differentially expressed at this developmental stage. Each bubble represents a GO term, categorized based on Biological Process, Cellular Component, or Molecular Function. The Gene Ratio represents the proportion of DEGs associated with a specific GO term relative to the total number of input DEGs. The Counts indicate the absolute number of DEGs mapped to each GO term in the database. Bubble size corresponds to the number of DEGs (Counts, indicating the absolute number of DEGs mapped to each GO term in the database.), while color intensity reflects the statistical significance (adjusted p-value) of enrichment. Enrichment significance was determined using the Benjamini-Hochberg procedure with a false discovery rate (FDR) threshold of < 0.05. The Gene Ratio represents the proportion of DEGs associated with a specific GO term relative to the total number of input DEGs. (TIF) [file pgen.1011948.s009.tif]

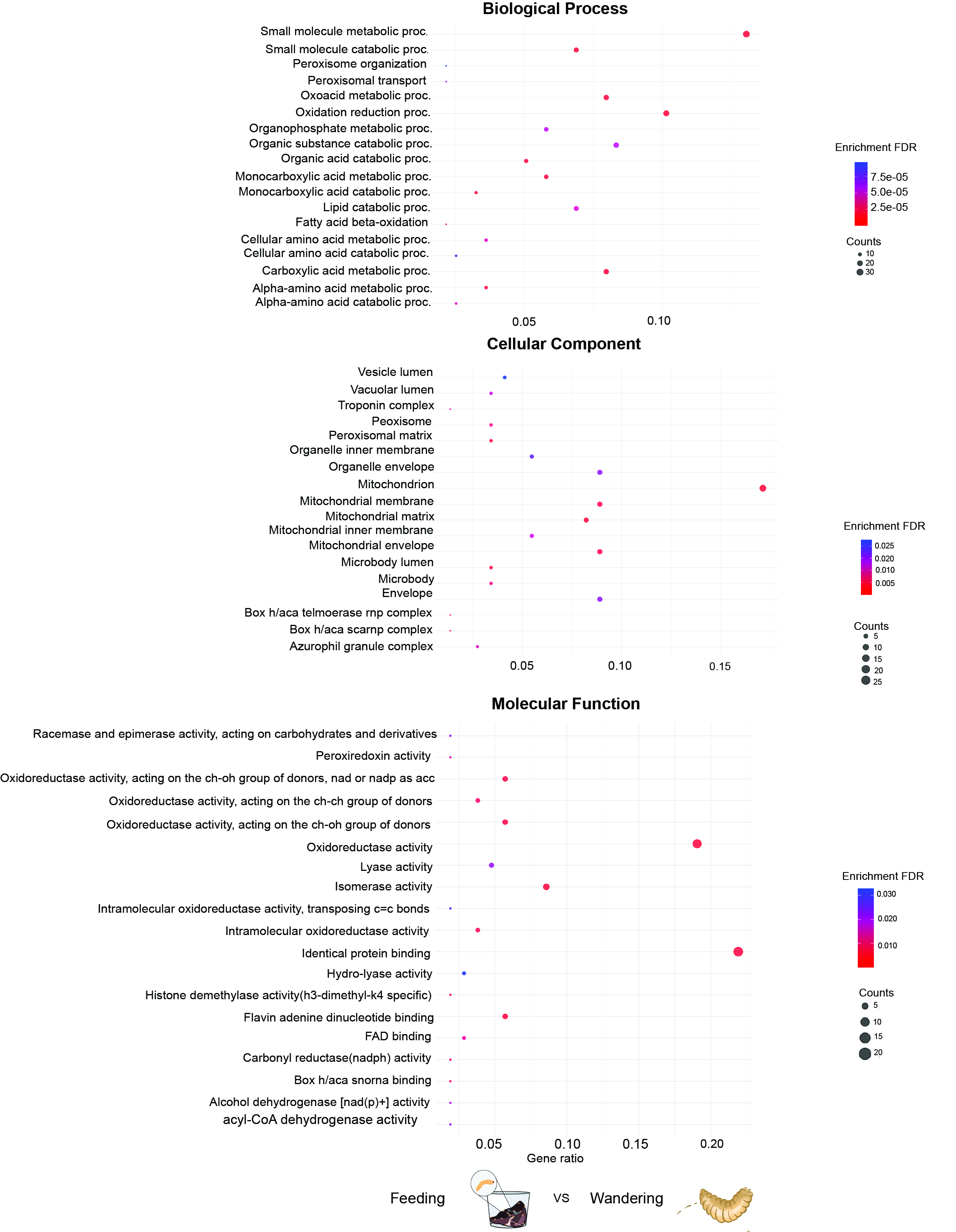

Supplement: S8 Fig — This analysis highlights the most significantly enriched GO terms among genes differentially expressed between feeding and wandering behaviors. Each bubble represents a GO term, categorized based on Biological Process, Cellular Component, or Molecular Function. The Gene Ratio represents the proportion of DEGs associated with a specific GO term relative to the total number of input DEGs. The Counts indicate the absolute number of DEGs mapped to each GO term in the database. Bubble size corresponds to the number of DEGs (Counts, indicating the absolute number of DEGs mapped to each GO term in the database.), while color intensity reflects the statistical significance (adjusted p-value) of enrichment. Enrichment significance was determined using the Benjamini-Hochberg procedure with a false discovery rate (FDR) threshold of < 0.05. The Gene Ratio represents the proportion of DEGs associated with a specific GO term relative to the total number of input DEGs. (TIF) [file pgen.1011948.s010.tif]

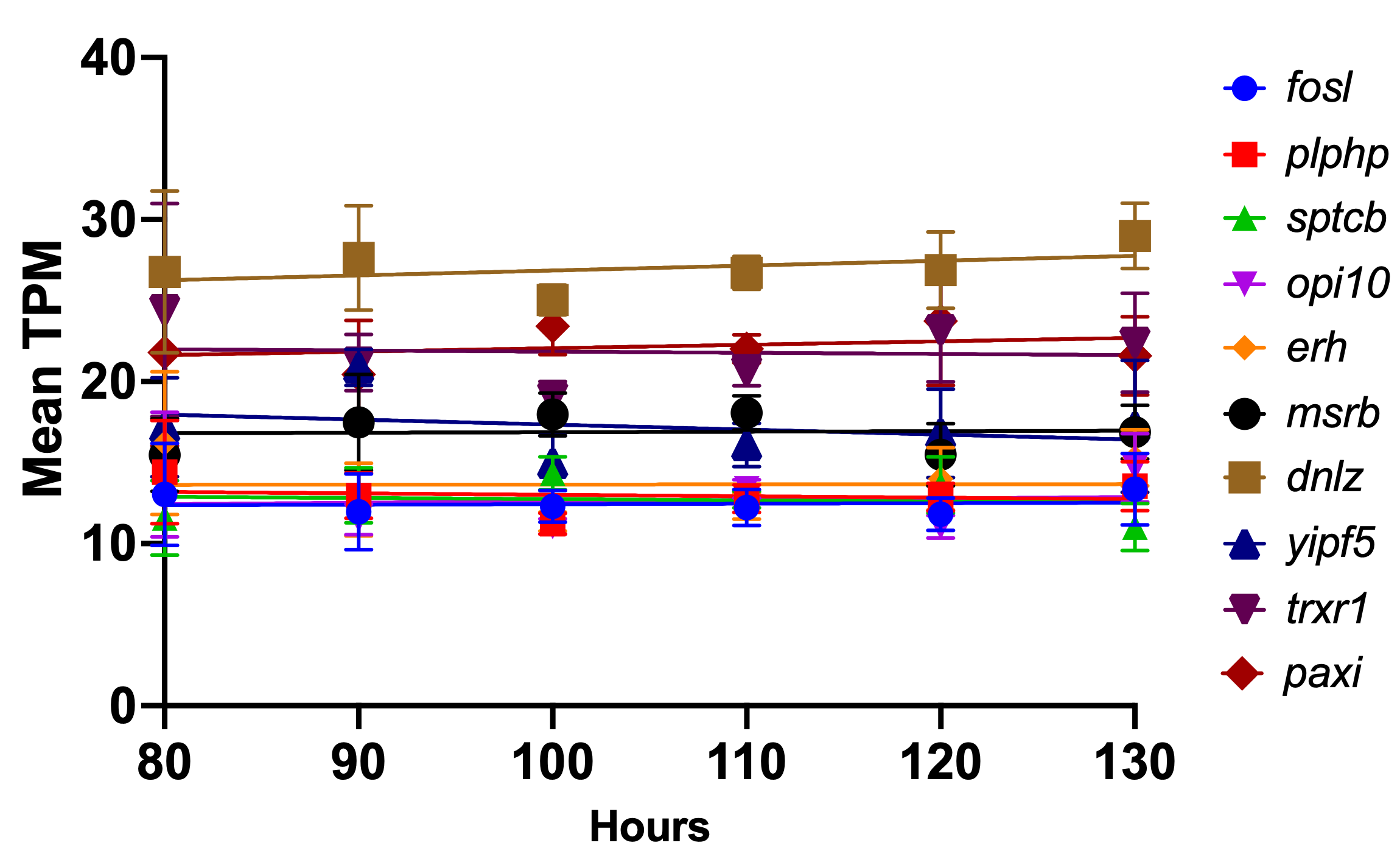

Supplement: S9 Fig — Linear regression model showed 10 housekeeping genes expressed over time (80 h ~ 130 h) at transcripts per millions (TPM) values (s < -2, all values greater than 0). (TIFF) [file pgen.1011948.s011.tiff]
